# Implications for Social Impact of Dialogic Teaching and Learning

**DOI:** 10.3389/fpsyg.2020.00140

**Published:** 2020-02-05

**Authors:** Rocío García-Carrión, Garazi López de Aguileta, Maria Padrós, Mimar Ramis-Salas

**Affiliations:** ^1^Faculty of Psychology and Education, University of Deusto, Bilbao, Spain; ^2^IKERBASQUE Basque Foundation for Science, Bilbao, Spain; ^3^Department of Curriculum and Instruction, University of Wisconsin–Madison, Madison, WI, United States; ^4^Faculty of Education, University of Barcelona, Barcelona, Spain; ^5^Department of Sociology, Faculty of Economics and Business, University of Barcelona, Barcelona, Spain

**Keywords:** dialogic teaching and learning, social impact, social improvements, social cohesion and education, dialogic education

## Abstract

The science of dialogic teaching and learning has especially flourished over the last four decades across age-groups, cultures, and contexts. A wide array of studies has examined the uniqueness of dialogue as a powerful tool to lead effective instructional practices, transform the socio-cultural context and people’s mindsets, among many others. However, despite the efforts to extend the benefits of this approach, certain difficulties exist which have hindered the consolidation of dialogic pedagogies in the classroom. This review discusses the implications for social impact of the scientific developments on dialogic teaching and learning. Particularly, an overview of the state of the art on dialogic education is presented. Social improvements in academic attainment and social cohesion are some of the fundamental issues discussed. Those are especially relevant to address crucial needs in education and solve some of the most pressing social problems. A communicative mix-methods approach emerges as one of the critical aspects of this field of research in educational psychology to achieve social impact. Some limitations, such as teachers sustaining different forms of monologic discourse, and challenges for a broader impact are discussed in this review.

## Introduction

Consistent with the dialogic turn in our societies, educational psychology has been affected by this “dialogic shift” that has inspired the advancements in the science and practice of dialogic teaching and learning (Racionero and Padrós, 2010). Educational psychology made a turn in how individual and cognitive elements were understood, including broader factors in the learning process: from a focus on mental schemata of previous knowledge to a focus on culture, intersubjectivity, and dialogue as crucial for learning and development (Bruner, 1996; Lee, 2016). This shift has influenced a growing interest by researchers in the fields of educational psychology, sociology, anthropology, and linguistics to study the social processes of learning and development, as well as teachers’ acceptance of the importance of classroom interactions (Mercer and Dawes, 2014). As a result, research on classroom dialogue and academic learning has grown considerably over the past 40 years (Howe and Abedin, 2013) and especially within the last decade (Resnick et al., 2015).

This shift in educational psychology has influenced multiple advancements in the creation of scientific knowledge on the diversity of instructional practices based on dialogic teaching and learning which have contributed to several improvements: developing language and communication skills (van der Veen et al., 2017; Teo, 2019); promoting critical thinking and reasoning (Mercer et al., 1999; Teo, 2019); learning science and mathematics (Soong and Mercer, 2011; Díez-Palomar and Olivé, 2015; Alexander, 2018); boosting social inclusion and democratic values such as solidarity and friendship (Valero et al., 2017; Villardón-Gallego et al., 2018; Rios-Gonzalez et al., 2019); or empowering students to become agents of social change (García-Carrión and Díez-Palomar, 2015), among others.

Similarly, different methodologies have been recently developed in order to assess the impact of dialogic teaching and learning, as discussed in the Oxford Research Encyclopedia of Education (Wegerif, 2019). Due to the ambivalence derived from the multiple perspectives that inform meaning emerging in dialogism, assessing the impact of dialogic education can be complex. Therefore, particular methods that respond to the challenges that traditionally used monologic assumptions suppose – such as those used by government proxies and assessment interventions – have been developed. Among these methods, Flecha (2000) puts forward the “communicative method” of evaluation which – in line with dialogism claims – builds on the active engagement of the participants throughout the research process. Wegerif et al. (2017) proposed a type of evaluation in the form of written dialogue combining both “outside” views with “inside” ones, where the former is grounded in statistical sources and the latter in more qualitative insights. The abovementioned complexity of assessing the impact of dialogic education – in terms of, for instance, the extent to which certain ideas are being held down – claims the need for more than indicators such as success on standardized tests. Several authors have collected consistent evidence of the impact of dialogic education to meet curriculum goals including reasoning and also intelligence (Resnick et al., 2015).

Joint efforts between researchers and teachers have situated the use of dialogue at the center of educational classroom practices and research methodologies in different countries, school contexts, cultural groups, and educational levels (Hennessy et al., 2016; Teachman et al., 2018; Vrikki et al., 2019a). The use of dialogic interactions in the classroom has showed to create more opportunities for extended discourse and, consequently, it seems to be more beneficial for language development compared with non-dialogic interactions (Snow, 2014). However, the prevailing form of teacher–student interactions continues to be the traditional initiation–reply–evaluation (IRE) structure, in which the teacher initiates by posing a question looking for a preferred answer, the student responds, and the teacher evaluates the answer. Mehan and Cazden (2015) note that the classrooms which have followed this pattern have excluded many minority students, as it does not encourage them to actively participate in the classroom talk. Similarly, the initiation–response–feedback (IRF) format, originally recorded by Sinclair and Coulthard (1975), has been reported to be a common practice in classrooms worldwide (Nystrand et al., 1997; Wells and Arauz, 2006). This has been conveyed by observational studies by Howe and Abedin (2013), who note that the most effective forms of productive classroom dialogue are not as strongly rooted in daily classroom practice. Indeed, in spite of all the efforts to transfer the evidence on the benefits of dialogic teaching and learning to the classrooms, dialogism still encounters many barriers in the school setting, hindering a broader and deeper potential social impact of dialogic education. Some of these barriers might come from teachers who follow the traditional classroom ground rules which sustain different forms of monologic discourse (Mercer and Howe, 2012), or teachers’ tension between giving students freedom to interact with each other and delivering curriculum goals (Lyle, 2008; Howe and Abedin, 2013).

Consequently, the traditional forms of monologic discourse are still preventing many children from benefitting from the productive forms of dialogue and interaction that can “ensure inclusive and equitable quality education and promote lifelong learning opportunities for all” (United Nations Economic and Social Council, 2019). But if educational psychology aims at reaching social impact, it must tackle the challenge to counteract those practices that are excluding many children from quality education. Providing evidence-based knowledge to obtain quality education for all is one of the foundations to create sustainable development. Indeed, the social impact of science refers to the achievement of social improvements aligned with the needs and goals of our societies, after disseminating and transferring research results (Reale et al., 2018). Thus, the science of dialogic teaching and learning should be relevant and effective in practice to ultimately lead to the social improvements required to provide all children with their inherent right to quality education. This is in line with this research topic and with the growing claim that the whole spectrum of sciences faces to demonstrate their public value.

This review argues that, although the scientific knowledge generated on dialogic teaching and learning during the last decades has contributed improvements which have opened pathways toward the achievement of the Sustainable Development Goals in education (SDG4-Quality Education), its implications for social impact have not been analyzed and developed in depth yet. This review aims at facilitating the theoretical discussion by making visible the existing implications and possibilities of educational research to contribute to the social impact of educational psychology and, in particular, of dialogic teaching and learning. Nonetheless, this attempt at exploring the social impact of the science of dialogic teaching and learning acknowledges the limitations the field has encountered for a more robust implementation of dialogic education in the classroom. To this end, the review discusses relevant works of the research line on dialogic education and their contributions to society. It shows two kinds of social impact and presents forms of measuring such impact to share it with the scientific community and put it at the disposal of society in order to keep moving forward on these advancements.

## Navigating a Dialogic Approach in Educational Psychology

Literature in the social sciences in general and in education in particular is reporting that dialogue has become essential in human relationships and actions in order to reach understanding and consensus among people (Habermas, 1981/1984). Grounding human relationships and actions on dialogue and interaction gives more agency to all individuals, instead of only to the ones who are in a power position, as it questions traditional hierarchies of power. As a result of the dialogic turn, dialogism is more and more present in every space conveying human relationships and actions, such as homes, the workplace, or classrooms, to name a few (Freire, 1970; Bakhtin, 1986).

Dialogue had already been one of the key elements in Vygotsky’s theory of cognitive development, which set the roots for educational psychology as we conceive it today, placing social interaction at the center of the learning and development processes (Vygotsky, 1978). With his contributions, research in the field of educational psychology shifted from studying children’s cognitive development as intra-mental activity to analyzing it as inter-mental activity, acquiring a sociocultural approach (García et al., 2010; Mercer and Howe, 2012; Littleton and Mercer, 2013). This is a fundamental Vygotskian concept that established our current understanding on the fact that language is the most important tool to think, learn, and develop, which takes place first at the social level and then at the individual one (Vygotsky, 1978). In other words, language serves as both a cultural (promoting *intermental* activity) and a psychological (promoting *intramental* activity) tool through which children *interthink*, that is, think and create meaning together, achieving higher mental functions which are central in cognitive development (Vygotsky, 1978; Mercer, 2000; Wells and Arauz, 2006). There is consensus on understanding that the social interactions that children have, both with their peers and with other adults, are crucial for their development and academic outcomes (Mercer and Howe, 2012).

Within this line of research, scholars have had different focuses of analysis from which to study dialogue and its impact on education. In what follows, some of the main perspectives placing dialogue at the center of their analysis are reviewed in order to highlight the contributions they have made to the theoretical discussion around the role of dialogue in teaching and learning. It will be made clear that, while some scholars direct their attention to the presence of dialogue in the teaching practice, others consider the relevance of dialogue as a tool for collective thinking in the classroom, and yet others are concerned with the elements that promote the creation of spaces that facilitate dialogic action.

### Dialogic Teaching

One of the proposals studied and developed to advance scientific knowledge and progress in this regard is dialogic teaching, which aims at using talk in effective ways for children’s learning and development. Several authors, such as Nystrand et al. (1997); Wells (1999), Alexander (2008); Resnick et al. (2015), or Mercer (1995) have been influential for the development of dialogic teaching. Such authors argue for the need to engage teachers and students in dialogue for the construction of knowledge and the understanding of the curriculum content, instead of knowledge and curriculum content being transmitted from teachers to students. Dialogic teaching thus moves away from the traditional teacher–student question and answer pattern to a dialogue propelled by teachers seeking to improve students’ learning and understanding (Alexander, 2008). In a comparative study on classroom talk in different countries, Alexander (2001) observed that in some schools, particularly in Russia, teachers used dialogue to engage students in questions and answers to develop their thinking. Influenced by Bakhtin’s (1986) idea that “if an answer does not give rise to a new question from itself, it falls out of the dialogue” (Bakhtin, 1986), he has contributed evidence on the dialogic approach to teaching to involve students in questions and answers with their peers and teachers in order to explore new thoughts and ideas (Wegerif, 2019). In his latest study, Alexander (2018) conducted a randomized control trial of an intervention of dialogic teaching which aimed at maximizing the benefits of classroom talk to promote students’ engagement and learning. As a result of this large-scale study, data indicated that after 20 weeks participating in the study, students in the intervention group, whose teachers had received a more dialogic training, showed a 2-month advancement in English, Mathematics, and Science tests compared to the control group, whose teachers used traditional (Alexander, 2018).

The aim of dialogic teaching is to maximize the potential of the teacher–student interactions in order to attain the best educational outcomes and improvements for all children. Dialogic teaching encourages students to think and question ideas, to explore new points of view, and to construct knowledge in dialogue with their peers and with teachers (Alexander, 2008). Resulting from this, research has shown that classrooms become more inclusive, as all students are invited to increase their participation and take an active and meaningful role in the discussions (Lyle, 2008; Mercer and Howe, 2012). Through fostering dialogic interactions in the classroom, dialogic teaching not only promotes wider and deeper thinking and learning among students, but it transforms classroom relationships, readjusting the traditional power relation between teachers and students (Teo, 2019). The ground of this approach relies on dialogue based on democratic values, through which students work together to reach understanding and complete tasks, moving forward in their thinking and reasoning. Although more research is needed to gather the social impact of dialogic teaching, recent research has provided evidence which supports the idea that the way in which teachers use dialogue in the classroom matters for children’s learning (Mercer, 2019). For instance, Howe et al. (2019) observed and recorded teacher and student dialogues in 72 diverse classrooms, finding that students whose teachers promoted classroom dialogue involving many students achieved better results in English and mathematics than the students whose teachers did not encourage such dialogue.

Importantly, fine-grained analysis of dialogic interactions has shown that not all kinds of dialogue in the classroom promote children’s higher levels of thinking and understanding. Therefore, Mercer and Howe (2012) propose a distinctive use of the concept of dialogue, not to refer to any kind of talk, but rather to a “form of conversation in which the ideas of the various participants are heard, taken up and jointly considered” (p. 14). Due to the long tradition and prevalence of the monologic IRE and IRF models in the classrooms, it is often the case that teachers are still the ones who direct the dialogues by making questions, pointing out who should speak, or being the only ones assessing the quality of the interactions (Mercer and Howe, 2012). This leaves little room for students to truly interact in a beneficial way, which is why research has emphasized the importance of teachers’ role to scaffold students’ development by encouraging interactions through which they exchange their ideas and thoughts in a truly dialogic, collaborative, and productive way (Alexander, 2001; Mercer, 2013). Building on his earlier work, Alexander (2018) provides a dialogic teaching framework where he discusses dialogic teaching not as a single definition but as “an interlocking set of permissive repertoires through which, steered by principles of procedure, teachers energize their own and their students’ talk” (Alexander, 2018, p. 561). The five principles underpin teacher–student interactions and may foster a dialogic pedagogy in the classroom (Supplementary Table 1).

### Using Language to Think Collectively

Another one of the most relevant schools of thought concerning the centrality of dialogue in education has focused precisely on the analysis of children’s dialogue aiming to shed light on the type of interactions that effectively trigger higher levels of thinking and understanding. Mercer (2019) has been studying talk and how children and adults use it in the most effective and productive ways to “share information, guide learning, develop joint understanding, critically evaluate ideas and find creative solutions to life’s burning issues” for decades (p. 8). To better understand the social nature of human cognition, as well as to contribute improvements to children’s learning and to teaching practices, he is devoted to providing evidence that supports the view that learning and development, as well as creativity, are best attained in collaboration (Mercer, 2019). Being aware that not all classroom interactions lead to children’s development and learning, Edwards and Mercer (1987); Mercer (1995), and Mercer and Dawes (2014) have studied different kinds of classroom talk in depth, providing repertoires of practices that lead to maximizing children’s learning and development through particular types of dialogue and interactions (Vrikki et al., 2019b). As a result, three types of talk have been identified among students’ interactions: disputational, cumulative, and exploratory talk (Littleton and Mercer, 2013) with different impacts on children’s learning process. Disputational talk was found to be the least productive and collaborative one, as it refers to interactions where there is disagreement, competitiveness, and individualized decision-making. As concerns cumulative talk, although research shows that it entails a broader acceptance of others’ ideas than disputational talk does, it still lacks the critical evaluation of these ideas. Unlike the two former ones, exploratory talk is the collaborative sort of dialogue through which students exchange and challenge each other’s ideas and critically, but constructively, analyze them. Evidence shows that it is the most productive and effective form of student interaction among the three identified ones, contributing to improvements in students’ attainments in several domains including mathematics, science, and problem-solving (Vrikki et al., 2019b).

Exploratory talk is characterized by a critical engagement with each other’s ideas to ultimately achieve an agreement (Vrikki et al., 2019b). In exploratory talk, students are not only participating in a collaborative activity, but they are *interthinking* (Mercer, 2000). Therefore, this kind of interaction triggering collective thinking is essential for students in order not only to communicate with each other, but to understand other people’s minds, help each other, reason, create knowledge, and solve problems together (Mercer, 2013). However, in spite of the positive impact collected, Mercer’s analysis of different classroom interactions shows that exploratory talk has been observed to be used with less frequency (Mercer and Howe, 2012; Vrikki et al., 2019b). These authors explain that this is due to a set of conversational ground rules which are expected to be followed according to normal school culture, such as the monologic discourse in which teachers take up almost all classroom interactions (Mercer and Howe, 2012). As the authors point out, “research has shown that adherence to these ground rules limits the potential value of talk among teachers and students” (Mercer and Howe, 2012, p. 17). Barriers for interacting in exploratory talk in the classroom have been encountered by both teachers and students. On the one hand, teachers face tensions between providing students with freedom to discuss their ideas and views and their need to meet the curriculum goals; on the other hand, students also find it difficult to challenge each other’s ideas (Howe and Abedin, 2013).

To counter these obstacles, and in line with dialogic teaching, Mercer also studies teacher–student interactions which can scaffold students’ achievement of exploratory talk. In this sense, teachers (or other adults in the classroom) are prompted to take the responsibility of guiding students in challenging their classmates’ ideas and proposing alternative hypotheses, urging them to develop arguments and reasoning (Mercer, 2013). In so doing, exploratory talk is granted with ground rules which will make this kind of talk truly dialogic and collaborative, by means of incorporating all students’ voices and points of view in order to discuss them and ultimately reach an agreement on the problem solving (Knight and Mercer, 2015). Such ground rules are (Mercer et al., 1999, p. 98–99):

(1) all relevant information is shared,
(2) the group seeks to reach agreement,
(3) the group takes responsibility for decisions,
(4) reasons are expected,
(5) challenges are accepted,
(6) alternatives are discussed before a decision is taken, and
(7) all in the group are encouraged to speak by other group members.

When children are encouraged to follow these ground rules, they get directed to using talk in a collaborative and productive way in order to complete tasks together. Their thinking and reasoning skills are expanded when, in engaging in this kind of dialogue, they challenge each other’s ideas at the same time that they provide arguments to support theirs in order to complete the activity. This dialogic practice triggered or facilitated by teachers, peers, or other adults focuses on the development of a particular type of talk with its own rules to be followed in order to guarantee the quality of the dialogue.

### Advancing Toward a Dialogic Space

Yet other approaches to dialogic education place the focus of attention not on the elements of the very dialogue which will promote a particular impact in the learning process, but rather on the social activity that facilitates dialogue. When students are engaged in truly collaborative activities in which they need to interact to discuss their ideas and construct common knowledge, dialogue is not just the means through which the students will complete the task, but it is also the goal of the collaborative activity and, in all, of education itself (Wegerif, 2011). In this vein, Wegerif (2011) developed the concept of the dialogic space applied to the interactive communications technology (ICT). By space he does not mean physical space, but rather the social activity of thinking and acting together (Mercer et al., 2010; Wegerif, 2011). The dialogic space therefore conveys the ground for shared thinking and reasoning to reach higher levels of learning and understanding and create new meanings. It is also the shared space through which students and teachers learn from each other by seeing “the task through each other’s eyes” (Wegerif, 2007, in Mercer et al., 2010).

Wegerif (2011) argues that human thinking is essentially dialogic. As has been previously mentioned, dialogism is more and more present in our everyday lives; we are constantly sharing thoughts, knowledge, different viewpoints which, in dialogue, can serve us to develop our own – and our communities’ – arguments and ideas and to advance in the construction of new knowledge. Thinking and reasoning necessarily requires listening to each other’s ideas and learning from different perspectives in dialogue with each other and with ourselves (Wegerif, 2011). Therefore, education needs to convey dialogic spaces to prepare children for these dialogues in order to advance their learning, thinking, and development, contributing not only to their success at school, but also in new contexts throughout their lives.

As a result of these advancements in the knowledge generated on dialogic education, Cambridge Educational Dialogue Research Group (CEDiR) was launched in the University of Cambridge in 2015 and is currently co-led by Sara Hennessy and Rupert Wegerif. The group’s aim is to conduct cross-disciplinary research that contributes to the development of educational dialogue and its impact in theory, practice, and policy.

### Theory and Practice of Dialogic Learning

Devoting his analysis not only to the theoretical advances of dialogic learning but also to its most successful practice, Flecha (2000) has conducted research to study the transformative impact of dialogue in different spheres of society. The work of Flecha (2000) provides all children – regardless of their origin, culture, or background – with the same opportunities to participate in dialogic spaces that promote their learning and development. He has done so, on the one hand, through his theoretical development of the seven principles of dialogic learning; and, on the other hand, through research evidence on the educational actions which promote dialogic learning and improve learning and development. Through dialogic learning, children become the protagonists of their own learning process by engaging in dialogues with peers, teachers, and other volunteering adults who help them reach higher levels of thinking, reasoning, and understanding which they would not be able to attain on their own.

Flecha (2000) has devoted research to studying the transformative impact of dialogue in different spheres of society. The seven principles of dialogic learning provide conceptual guidelines to facilitate the process of in-depth learning-related social transformations (Supplementary Table 2).

In line with previously discussed authors, dialogic learning grants students opportunities to engage in interactions which lead them to higher levels of reasoning, thinking, and development. This is done through the creation of dialogic spaces that put these principles into practice, like the dialogic literary gatherings (DLG). These are contexts where participants (who might be adults in literacy processes or school children) engage in a dialogue around the classical works of universal literature such as Cervantes’s *Quixote*, Joyce’s *Ulysses*, or Garcia Lorca’s *La Casa de Bernarda Alba*, to name only a few. Through the particular functioning of the DLG – all participants have the equal right to speak, the contributions are given value according to the argument they convey and not to an alleged hierarchy of participants, etc. – participants create new meaning about the particular literary work they are discussing. Flecha’s (2000) contributions have an extended impact, as students internalize the learning outcomes and transfer them onto their families, neighborhoods, and communities, becoming not only the recipients of profound transformations but also their very triggers in enlarged contexts (Soler, 2015).

In 2006, Flecha conducted the only EU-funded research project in the field of Socioeconomic Sciences and Humanities of the Framework Programmes for Research selected by the European Commission among the 10 examples of success stories (European Commission, 2011). The project studied and analyzed several successful educational actions (SEAs) throughout different European countries (Flecha, 2015). SEAs are evidence-based educational actions grounded on dialogic learning which have shown to achieve the best results in different contexts all over the world (Flecha, 2015). Therefore, SEAs provide all students, no matter where they come from, with the same opportunities for attaining excellent academic achievements and participating in transformations which overcome exclusion and many other barriers children in different contexts encounter.

## Social Impact of Implementing Dialogic Teaching and Learning in Schools

The demand for science to generate socially relevant knowledge that contributes improvements to society is becoming increasingly relevant in all scientific domains and social contexts (Reale et al., 2018). In spite of the limitations identified and introduced in this review, sufficient evidence has been provided showing the particular benefits for education – in at least two dimensions: academic achievement and social cohesion – of dialogic teaching and learning, thus contributing to generating the desired social impact. These dimensions are at the core of the targets defined by the United Nations Statistics Division Goal 4: Quality Education. Therefore, the goal has been to collect, systematize, and present evidence of this social impact from different European research projects, showing the improvements and benefits achieved with dialogic teaching and learning in the two dimensions mentioned here (Howe et al., 2019).

### Dialogic Education for Improving Academic Achievement

Accumulated evidence both from small-scale and large-scale studies has provided relevant evidence supporting dialogic teaching and learning as a key contribution to education. For example, a number of studies based on intervention programs for teaching children how to use dialogue in a productive and efficient way have also been found to achieve improvements in academic attainments in different subjects and skills, such as reasoning or math problem-solving (Mercer and Sams, 2006). Relevant evidence, both quantitative and qualitative, show that after participating in intervention studies based on dialogic teaching, children in the target groups increase the use of exploratory talk during group activities as opposed to the control groups (Mercer et al., 1999; Mercer, 2000). These results imply that, when children are taught how to use language in an effective way for collaborative activities, their participation in the dialogue increases, and so do their achievements, contributing to improvements in different subjects and skills (Mercer and Sams, 2006). A study carried out with 60 British Primary students revealed that after the 10 weeks that the dialogic teaching program lasted, children’s individual scores in the Raven’s Progressive Matrices showed greater gains in the students of the target group than those of the control group (Mercer et al., 1999). Although dialogic interaction studies have traditionally focused on small group interactions among students, other more recent large-scale studies have focused on the impact of interactions between teachers and students on the latter’s performance.

The benefits associated to these interventions are especially relevant for children with the least resources, who live in low socioeconomic status (SES) backgrounds when acquiring and developing, for example, literacy skills (Levy et al., 2018). Indeed, academic attainment is one of the key elements which can help them break the barriers imposed to them and overcome such exclusion, defying deterministic theories which have long been proven wrong. Existing evidence shows the relevant implications that different practices and interventions grounded in dialogic education are providing to improve children’s educational outcomes, particularly important in the case of the most vulnerable groups living in poverty (Lampert et al., 2019). In this line, research on dialogic teaching indicated that, after the 20-week large-scale intervention with 2493 4th grade students, those in the target group achieved an average of a 2-month greater progress in English and science than the control group, and a 1-month progress in mathematics (Alexander, 2018). This is particularly important in the case of students qualifying for free school meals (a standard measure for poverty in the United Kingdom) who attained a 2-month progress in mathematics (Alexander, 2018).

Alexander’s (2018) and Howe et al.’s (2019) studies are in line with another key large-scale research project funded by the European Commission’s Framework Programme^1^, which after conducting 26 longitudinal case studies in 7 European countries, presented a set of SEAs based on dialogic learning which achieved improvements in academic outcomes in a range of diverse schools and contexts (Flecha, 2015). These results have been further analyzed during the last decade through other EC-funded projects that study the elements facilitating the implementation and transfer of SEA to new contexts in different EU countries.

Some of the main results of the analyses conducted throughout such research point that SEA have contributed to high quality education at the different educational levels and contexts they have been implemented in, from early childhood education to adult education or out-of-home child care centers (Pulido, 2015; Aubert et al., 2017; Garcia Yeste et al., 2018). For instance, research has shown that interactions based on egalitarian dialogue operating in small heterogeneous groups of students known as interactive groups (IGs) boost children’s learning of mathematics, particularly in terms of mathematics understanding and problem-solving, contributing to the improvement in mathematics performance (Díez-Palomar and Olivé, 2015; Flecha, 2015; García-Carrión and Díez-Palomar, 2015). The evidence shows that the advancements in mathematics skills due to participating in IG also foster an increase in students’ self-confidence, self-efficacy, and a positive attitude toward mathematics (Díez-Palomar and Olivé, 2015; García-Carrión and Díez-Palomar, 2015; Díez-Palomar et al., 2018). In a similar vein, an experimental study conducted to analyze children’s productivity when working in groups to solve math problems showed that the children in the target group engaged in collaborative, enthusiastic, and productive ways more than the control group and, as a result, achieved greater improvements in their attainments in mathematics (Mercer and Sams, 2006).

On the other hand, DLG, another SEA promoting interactions based on egalitarian dialogue, have been reported to enhance students’ reading skills, vocabulary acquisition, and knowledge of cultural and historical concepts (de Botton et al., 2014; Serradell, 2015). Research has found a shift in the teacher–student talk ratio in DLG from the monologic discourse to students’ participation taking up over 80% of classroom talk (Hargreaves and García-Carrión, 2016). However, the egalitarian dialogue upon which DLG are based does not only increase students’ talk ratio, but it also improves the quality of classroom interactions. Indeed, following the egalitarian dialogue principle of dialogic learning, all children have the same rights and opportunities to participate and provide their own ideas and opinions to the dialogue, supporting them with arguments rather than imposing them through power positions. This way, students are encouraged to develop argumentation and reasoning, as well as to question and counter-argue classmates’ ideas (Flecha and Soler, 2013; Serradell, 2015).

The social improvements generated as a result of implementing these dialogic learning-based actions have been found to benefit all students, including those with disabilities. A case study aimed at exploring the learning opportunities that these actions grant children with disabilities in special schools found that interactions among students became richer, and that instrumental learning, especially in mathematics, improved in these dialogic spaces (García-Carrión et al., 2018).

### Fostering Social Cohesion Through Dialogic Education

Classrooms based on dialogic teaching and learning have proven that teachers do not need to choose between fostering students’ academic achievements or social cohesion. Rather, evidence on some of the dialogue-based practices presented in the previous section shows that developments in instrumental learning, competences, and skills, when boosted through egalitarian dialogue, influence prosocial values such as solidarity and friendship (Villardón-Gallego et al., 2018); and that, at the same time, when such values are developed, instrumental learning and academic attainments are propelled. Therefore, one dimension promotes the other, and vice versa.

Dialogic learning environments, for instance IG, are one of the examples of how this reciprocal relationship between instrumental learning and prosocial behaviors occurs. Because the aim of IG is not only for children to complete the activities but for all of them to understand and solve them together, children are required to interact to help each other, to explain the activity to those who have not understood it. This overarching goal of IG builds dynamics of mutual support among peers: while children’s instrumental learning in different subjects is being promoted, the fact that the activities need to be completed in dialogic interactions boosts inclusion and democratic values such as solidarity, support, and friendship among the students who help each other to solve the activities (Aubert et al., 2017; Valero et al., 2017). Therefore, children do not learn these values by being talked about them, but by putting them into practice (Aubert et al., 2017; Valero et al., 2017). At the same time, learning those values by putting them into practice contributes to a deeper internalization of them. Moreover, the values they learn and internalize do not just stay inside the classroom, but students transfer them to other spaces such as the playground, the neighborhood, or the family (Aubert et al., 2017). Besides, this dialogic environment can be particularly beneficial for students with disabilities, who often suffer from exclusion and are discriminated against (García-Carrión et al., 2018). The egalitarian dialogue fostered in IG provides students with disabilities with the same opportunities as the rest of the students to participate and contribute to the group, thus promoting the rejection of the labeling commonly attached to these children (García-Carrión et al., 2018). The previously mentioned study on interactive environments in special schools contributed evidence that, besides the academic achievements, the students with disabilities who participated in IG constructed safe, solidary, and supportive relationships with their peers, promoting their social inclusion (García-Carrión et al., 2018).

Promoting behaviors and relationships based on values such as solidarity, peer support, and friendship has also been found to reduce school conflict (Aubert, 2015; Villarejo-Carballido et al., 2019). In particular, the dialogic model of conflict prevention and resolution – a community-based educational intervention – has shown to be fostering solidarity networks among students facing school conflicts, creating safer learning environments in which conflicts such as bullying or cyberbullying are decreased (Villarejo-Carballido et al., 2019).

Research on other dialogic spaces such as DLG, in which through the universal classics of literature students open up to each other about their lives, feelings, and experiences regarding some of humanity’s deepest issues portrayed in the classic texts has reported evidence that these dialogues promote respect, tolerance, and empathy, among others, toward one another. It is the case of Amaya, a Roma girl who used to suffer bullying from her classmates and, as a result, started getting disengaged in school activities. However, when she started participating in DLGs at school, her classmates’ (and her own) perceptions toward her were transformed when seeing the passion with which she got involved in the DLG, and they stopped bullying her (Aubert, 2015). These findings were reported in a study that used the communicative methodology, in which through the egalitarian dialogue established between the researcher and Amaya herself, they constructed her biography by reflecting turning points in her school trajectory (Aubert, 2015). On the other hand, the first quasi-experimental study on the impact of DLG on children’s prosocial behavior provided evidence that the experimental groups which participated in 10 weekly DLG sessions developed prosocial behaviors such as solidarity and friendship to a greater extent than the control groups, which maintained or even decreased such behaviors during the same period (Villardón-Gallego et al., 2018).

As dialogue has entered the classroom, the monologic discourse is being increasingly replaced by egalitarian interactions, providing students with high-quality education and agency to become the protagonists of their own learning process and social development. Although more efforts need to be made to overcome the limitations for a more propagated implementation of dialogic teaching and learning practices into the classrooms, findings reported so far show that the inclusion of the students’ voices in the teaching and learning process contributes to a greater social engagement, as it encourages them to take an active role in the classroom, to develop reasoning, and their own viewpoints (Alexander et al., 2017).

## Discussion, Limitations, and Further Research

The efforts and dedication of countless researchers in the field of educational psychology to provide answers and solutions to educational and social challenges have been consolidated over the last decades. In particular, the potential benefits of dialogic teaching and learning have been explored through a series of small-scale (Díez-Palomar and Olivé, 2015; Aubert et al., 2017; García-Carrión et al., 2018; Garcia Yeste et al., 2018) and large-scale studies (Mercer and Sams, 2006; Flecha, 2015; Alexander, 2018; Howe et al., 2019). Currently, we count with enough evidence supporting the dialogic approach to ultimately provide effective pedagogical responses in which no child is excluded from classroom discourse.

This manuscript has discussed some of the studies and highly renowned contributions in the field with the aim of gathering their potential social impact to advance toward an inclusive and equitable quality education for all. We argue that the science of teaching and learning can play an important role in that ambitious endeavor. Indeed, evidence regarding the improvements achieved in learning outcomes and social cohesion in schools offers an opportunity for practitioners and policymakers to make the most of the evidence reported for more than 40 decades. At the same time, the researcher’s focus needs to move beyond the ivory tower to address the current educational and social needs (Tierney, 2013).

These improvements are persistently included in all public definitions of desirable horizons to be attained by our societies, as it was the case in the past Europe 2020 Agenda, where Education was one of the five targets defined, and now in the current Sustainable Development Goals of the United Nations. In this sense, educational research needs to be directed to providing all children with the opportunity to achieve academic outcomes while developing values, serving them as tools for hopeful, successful futures. The studies reviewed in this manuscript reveal that the science of dialogic teaching and learning has a potential for achieving such impacts.

Nonetheless, in spite of the progress made toward the social impact of dialogic teaching and learning, it has still not been expanded to all classrooms. This is clearly a limitation for measuring the potential social impact of this approach. In fact, a series of barriers have been encountered and discussed above hindering a more robust propagation of dialogicity. Particularly, Mercer and Howe (2012) highlight the school culture in which teachers dominate classroom dialogue as one of the obstacles for the implementation of dialogic education. They claim that traditional ground rules by which teachers are the only ones who, among other things, decide who should speak, make the questions, or evaluate students’ comments, are still prevalent in many classrooms, therefore leaving little space for effective and valuable talk among students (Mercer and Howe, 2012). In addition to these power relations between teachers and students, many teachers do not have the required skills for planning effective classroom dialogue, thus decreasing its potential to benefit children’s learning (Lyle, 2008). In a similar vein, Howe and Abedin (2013) point that teachers often find it difficult to promote exploratory talk among students as they find a tension between letting children discuss and explore each other’s views freely while monitoring what students are saying and introducing target knowledge in the discussion. In this sense, little guidance is given to teachers on how to effectively organize group work (Howe et al., 2007).

Students also find their own barriers for engaging in effective classroom dialogue, as many have experienced traditional forms of classroom talk such as the IRE or IRF models and, therefore, are not used to interacting among each other in a way that is not constrained by the teacher. In order to work effectively in groups, students need to learn and understand the new ground rules for effective classroom dialogue, as well as the value of effective dialogue for learning (Mercer and Howe, 2012). However, despite the evidence provided on the benefits of group work, proving to be an effective pedagogy, it is still a neglected art in many classrooms, and teachers in England do not use it enough, favoring more traditional classrooms (Galton and Hargreaves, 2009).

Research methodologies should tackle the problem aiming at obtaining socially relevant results. For that purpose, including the voices of teachers and students, as well as other end-users from the education community, can further contribute to the overcoming of the limitations and challenges they face in the implementation of dialogic teaching and learning. In line with the dialogic turn of our societies, some of the research approaches exploring the impact of dialogic teaching and learning are developed through the communicative methodology, an approach that places dialogue with the participants in a research process at its very core (Gómez et al., 2019). The involvement of teachers, students, and relatives in discussions on the results throughout the whole research process contributes to the prevention of bias on the interpretations of data and, thus, to better responding to their real needs. This involvement also facilitates the production of early improvements for the end-users, improves the credibility of the results, and expands the dissemination of dialogicity in formal and informal ways to a wider range of actors (other teachers, families, students).

Involving families and teachers in the educational theories and practices which have been proven to have an impact in other contexts gives them the opportunity, as well as their right, to demand such evidence to be put into practice in their educational communities. In the case of educational psychology, particularly of research on dialogic teaching and learning, scientific contributions might be critical for generating improvements in different settings and collecting evidence of such improvements to eventually extend and replicate them across contexts. This dialogic process implemented throughout all the research process, from providing participants with evidence of dialogic education in other contexts to discussing with them current challenges and possibilities for its implementation in their own context, allows both scientists and end-users to co-create new knowledge which will benefit the communities themselves and can contribute to social impact. It is essential to co-create knowledge with teachers and families to boost the overcoming of monologic discourse-based practices and increase the actual praxis of dialogic spaces and interactions that foster learning opportunities for all.

Although this review has discussed the implications for social impact of the science of dialogic teaching and learning, efforts must continue to be made in order to assess such impact. Assessing and evaluating the impact of dialogic education is still a complex task that, however, needs to be done. The challenges that lay ahead for assessing social impact (time lapse for achieving or extending that impact, or attribution of improvements to a specific research, for instance) are shared with all other scientific fields. Following the EC Report on *Monitoring the impact of EU Framework Programmes* (van den Besselaar et al., 2018), new assessments need to avoid the confusion between dissemination or transference and social impact, as the mere use of knowledge does not necessarily involve positive effects. In this vein, and following the indicators of the mentioned Report, researchers in educational psychology will need to gather evidence of the effects of the use of scientific results on tackling the SDG4, as well as of their replicability and sustainability. While there is an assumption that complete social impact is achieved in a long term, the examples that we have presented in this article support the standpoint that social impact can already be achieved from early stages and even during the lifespan of a project. In fact, the very nature of dialogic teaching and learning research, many times undertaken in close relationship with schools and end-users, allows to have both quantitative and qualitative evidences of the actual development of these dialogic practices. Even if these evidences are from small samples, understanding the link between research, research use, and social impact achieved will enhance the opportunities of scaling up the implementation of dialogic education.

Future research should therefore focus on advancing tools and methods to assess the improvements, sustainability, and replicability of dialogic teaching and learning in order to, on the one hand, advance in the visibility of this social impact. The prevailing trend of making the results of scientific research open to all citizenry is contributing to the expansion of the number of citizens from all walks of life who have access to research results, including the evidence of educational psychology that improves these same citizens’ and their children’s lives.

## Author Contributions

RG-C, GL, MP, and MR-S made substantial contributions to the conception of the manuscript, searching the literature, drafting the article, and revising it critically for important intellectual content, provided approval for publication of the content, and agreed to be accountable for all aspects of the work in ensuring that questions related to the accuracy or integrity of any part of the work are appropriately investigated and resolved.

## Conflict of Interest

The authors declare that the research was conducted in the absence of any commercial or financial relationships that could be construed as a potential conflict of interest. The reviewer VI declared a shared affiliation, with no collaboration, with several of the authors, MP and MR-S, to the handling Editor at the time of review.

## Footnotes

1 INCLUD-ED Project. Strategies for inclusion and social cohesion in Europe from education. 2006–2011. 6th Framework Programme. Citizens and Governance in a Knowledge-based Society. CIT4-CT-2006-028603. Directorate-General for Research, European Commission.

## References

[B1] Alexander R. J. (2001). *Culture and Pedagogy: International Comparisons in Primary Education.* Oxford: Blackwell.

[B2] Alexander R. J. (2008). *Towards Dialogic Teaching: Rethinking Classroom Talk*, 4th Edn York: Dialogos.

[B3] Alexander R. J. (2018). Developing dialogic teaching: genesis, process, trial. *Res. Pap. Educ.* 33 561–598. 10.1080/02671522.2018.1481140

[B4] Alexander R. J. Hardman F. C. Hardman J. (2017). *Changing Talk, Changing Thinking: Interim Report from the in-House Evaluation of the CPRT/UoY Dialogic Teaching Project.* York: University of York.

[B5] Aubert A. (2015). Amaya: dialogic literary gatherings evoking passion for learning and a transformation of the relationships of a Roma girl with her classmates. *Qual. Inq.* 21 858–864. 10.1177/1077800415614034

[B6] Aubert A. Molina S. Schubert T. Vidu A. (2017). Learning and inclusivity via interactive groups in early childhood education and care in the Hope school, Spain. *Learn. Cult. Soc. Interact.* 13 90–103. 10.1016/j.lcsi.2017.03.002

[B7] Bakhtin M. M. (1986). *Speech Genres and Other Late Essays. Trans. Vern W. McGee.* Austin, TX: University of Texas Press.

[B8] Bruner J. S. (1996). *The Culture of Education.* Cambridge, MA: Harvard University Press.

[B9] de Botton L. Girbés S. Ruiz L. Tellado I. (2014). Moroccan mothers’ involvement in dialogic literary gatherings in a Catalan urban primary school: increasing educative interactions and improving learning. *Improv. Sch.* 17 241–249. 10.1177/1365480214556420

[B10] Díez-Palomar J. de Sanmamed A. F. F. García-Carrión R. Molina-Roldán S. (2018). Pathways to equitable and sustainable education through the inclusion of Roma students in learning mathematics. *Sustainability* 10:2191 10.3390/su10072191

[B11] Díez-Palomar J. Olivé J. C. (2015). Using dialogic talk to teach mathematics: the case of interactive groups. *ZDM Math. Educ.* 47 1299–1313. 10.1007/s11858-015-0728-x

[B12] Edwards D. Mercer N. (1987). *Common Knowledge: The Development of Understanding in Classroom.* New York, NY: Methuen.

[B13] European Commission (2011). *Added Value of Research, Innovation and Science. MEMO/11/520.* Available at: http://europa.eu/rapid/press-release_MEMO-11-520_en.htm (accessed July 19).

[B14] Flecha R. (2000). *Sharing Words: Theory and Practice of Dialogic Learning.* Lanham, MD: Rowman & Littlefield.

[B15] Flecha R. (Ed.). (2015). *Successful Educational Actions for Inclusion and Social Cohesion in Europe.* Berlin: Springer.

[B16] Flecha R. Soler M. (2013). Turning difficulties into possibilities: engaging Roma families and students in school through dialogic learning. *Camb. J. Educ.* 43 451–465. 10.1080/0305764X.2013.819068

[B17] Freire P. (1970). *Pedagogy of the Oppressed.* New York: Continuum.

[B18] Galton M. Hargreaves L. (2009). Group work: still a neglected art? *Camb. J. Educ.* 39 1–6. 10.1080/03057640902726917 31412849

[B19] García R. Mircea T. Duque E. (2010). Socio-cultural transformation and the promotion of learning. *Rev. Psicodidactica* 15 207–222. 17399849

[B20] Garcia Yeste C. Gairal Casado R. Munté Pascual A. Plaja Viñas T. (2018). Dialogic literary gatherings and out-of-home child care: creation of new meanings through classic literature. *Child Fam. Soc. Work* 23 62–70. 10.1111/cfs.12384

[B21] García-Carrión R. Díez-Palomar J. (2015). Learning communities: pathways for educational success and social transformation through interactive groups in mathematics. *Eur. Educ. Res. J.* 14 151–166. 10.1177/1474904115571793

[B22] García-Carrión R. Molina Roldán S. Roca Campos E. (2018). Interactive learning environments for the educational improvement of students with disabilities in special schools. *Front. Psychol.* 9:1744. 10.3389/FPSYG.2018.01744 30283388 PMC6157546

[B23] Gómez A. Padrós M. Ríos O. Mara L. C. Pukepuke T. (2019). Reaching social impact through communicative methodology. Researching with rather than on vulnerable populations: the Roma case. *Front. Educ.* 4:9 10.3389/feduc.2019.00009

[B24] Habermas J. (1981/1984). *Theory of Communicative Action*: *Lifeworld and System: A Critique of Functionalist Reason*, Vol. 2 Boston, MA: Beacon Press.

[B25] Hargreaves L. García-Carrión R. (2016). Toppling teacher domination of primary classroom talk through dialogic literary gatherings in England. *FORUM* 58 15–26. 10.15730/forum.2016.58.1.15

[B26] Hennessy S. Rojas-Drummond S. Higham R. Márquez A. M. Maine F. Ríos R. M. (2016). Developing a coding scheme for analysing classroom dialogue across educational contexts. *Learn. Cult. Soc. Interact.* 9 16–44. 10.1016/j.lcsi.2015.12.001

[B27] Howe C. Abedin M. (2013). Classroom dialogue: a systematic review across four decades of research. *Camb. J. Educ.* 43 325–356. 10.1080/0305764X.2013.786024

[B28] Howe C. Hennessy S. Mercer N. Vrikki M. Wheatley L. (2019). Teacher–Student dialogue during classroom teaching: does it really impact on student outcomes? *J. Learn. Sci.* 28 462–512. 10.1080/10508406.2019.1573730

[B29] Howe C. Tolmie A. Thurston A. Topping K. Christie D. Livingston K. (2007). Group work in elementary science: towards organisational principles for supporting pupil learning. *Learn. Instruct.* 17 549–563. 10.1016/j.learninstruc.2007.09.004

[B30] Knight S. Mercer N. (2015). The role of exploratory talk in classroom search engine tasks. *Technol. Pedagogy Educ.* 24 303–319. 10.1080/1475939X.2014.931884

[B31] Lampert J. Ball A. Garcia-Carrion R. Burnett B. (2019). Poverty and schooling: three cases from Australia, the United States, and Spain. *Asia Pac. J. Teach. Educ.* 1–19. 10.1080/1359866X.2019.1602863

[B32] Lee C. D. (2016). Examining conceptions of how people learn over the decades through AERA presidential addresses: diversity and equity as persistent conundrums. *Educ. Res.* 45 73–82. 10.3102/0013189X16639045

[B33] Levy R. Hall M. Preece J. (2018). Examining the links between parents’ relationships with reading and shared reading with their pre-school children. *Int. J. Educ. Psychol.* 7 123–150. 10.17583/ijep.2018.3480

[B34] Littleton K. Mercer N. (2013). *Interthinking: Putting Talk to Work.* Abingdon: Routledge.

[B35] Lyle S. (2008). Dialogic teaching: discussing theoretical contexts and reviewing evidence from classroom practice. *Lang. Educ.* 22 222–240. 10.1080/09500780802152499

[B36] Mehan H. Cazden C. (2015). “The study of classroom discourse: early history and current developments,” in *Socializing Intelligence Through Academic Talk and Dialogue*, eds Resnick L. Asterhan C. Clarke S. (Washington, DC: American Educational Research Association). 10.3102/978-0-935302-43-1_2

[B37] Mercer N. (1995). *The Guided Construction of Knowledge: Talk Amongst Teachers and Learners.* Clevedon: Multilingual Matters.

[B38] Mercer N. (2000). *Words and Minds: How We Use Language to Think Together.* London: Routledge.

[B39] Mercer N. (2013). The social brain, language, and goal-directed collective thinking: a social conception of cognition and its implications for understanding how we think, teach, and learn. *Educ. Psychol.* 48 148–168. 10.1080/00461520.2013.804394

[B40] Mercer N. (2019). *Language and the Joint Creation of Knowledge: The Selected Works of Neil Mercer.* Abingdon: Routledge.

[B41] Mercer N. Dawes L. (2014). The study of talk between teachers and students, from the 1970s until the 2010s. *Oxf. Rev. Educ.* 40 430–445. 10.1080/03054985.2014.934087

[B42] Mercer N. Howe C. (2012). Explaining the dialogic processes of teaching and learning: the value and potential of sociocultural theory. *Learn. Cult. Soc. Interact.* 1 12–21. 10.1016/J.LCSI.2012.03.001

[B43] Mercer N. Sams C. (2006). Teaching children how to use language to solve maths problems. *Lang. Educ.* 20 507–528. 10.2167/le678.0

[B44] Mercer N. Warwick P. Kershner R. Staarman J. K. (2010). Can the interactive whiteboard help to provide “dialogic space” for children’s collaborative activity? *Lang. Educ.* 24 367–384. 10.1080/09500781003642460

[B45] Mercer N. Wegerif R. Dawes L. (1999). Children’s talk and the development of reasoning in the classroom. *Br. Educ. Res. J.* 25 95–111. 10.1080/0141192990250107

[B46] Nystrand M. Gamoran A. Kachur R. Prendergast C. (1997). Opening dialogue: understanding the dynamics of language and learning in the english classroom. *Language* 74 444 10.2307/417942

[B47] Pulido C. (2015). Amina, dreaming beyond the walls. *Qual. Inq.* 21 886–892. 10.1177/1077800415611691

[B48] Racionero S. Padrós M. (2010). The dialogic turn in educational psychology. *Rev. Psicodidáctica* 15 143–162.

[B49] Reale E. Avramov D. Canhial K. Donovan C. Flecha R. Holm P. (2018). A review of literature on evaluating the scientific, social and political impact of social sciences and humanities research. *Res. Eval.* 27 298–308. 10.1093/reseval/rvx025

[B50] Resnick L. B. Asterhan C. S. Clarke S. N. (2015). *Socializing Intelligence Through Academic Talk and Dialogue.* Washington, DC: American Educational Research Association.

[B51] Rios-Gonzalez O. Puigvert Mallart L. Sanvicén Torné P. Aubert Simón A. (2019). Promoting zero violence from early childhood: a case study on the prevention of aggressive behavior in Cappont Nursery. *Eur. Early Child. Educ. Res. J.* 27 157–169. 10.1080/1350293X.2019.1579544

[B52] Serradell O. (2015). Aisha, from being invisible to becoming a promoter of social change. *Qual. Inq.* 21 906–912. 10.1177/1077800415614030

[B53] Sinclair J. M. Coulthard M. (1975). *Towards an Analysis of Discourse: The English Used by Teachers and Pupils.* Oxford: Oxford University Press.

[B54] Snow C. E. (2014). Input to interaction to instruction: three key shifts in the history of child language research. *J. Child Lang.* 41 117–123. 10.1017/S0305000914000294 25023501

[B55] Soler M. (2015). Biographies of “Invisible” people who transform their lives and enhance social transformations through dialogic gatherings. *Qual. Inq.* 21 839–842. 10.1177/1077800415614032

[B56] Soong B. Mercer N. (2011). Improving students’ revision of physics concepts through ICT-based co-construction and prescriptive tutoring. *Int. J. Sci. Educ.* 33 1055–1078. 10.1080/09500693.2010.489586

[B57] Teachman G. McDonough P. Macarthur C. Gibson B. E. (2018). A critical dialogical methodology for conducting research with disabled youth who use augmentative and alternative communication. *Qual. Inq.* 24 35–44. 10.1177/1077800417727763

[B58] Teo P. (2019). Teaching for the 21st century: a case for dialogic pedagogy. *Learn. Cult. Soc. Interact.* 21 170–178. 10.1016/J.LCSI.2019.03.009

[B59] Tierney W. G. (2013). 2013 AERA presidential address: beyond the ivory tower: the role of the intellectual in eliminating poverty. *Educ. Res.* 42 295–303. 10.3102/0013189X13502772

[B60] United Nations Economic and Social Council (2019). *Special Edition: Progress Towards the Sustainable Development Goals Report of the Secretary-General.* Available at: https://undocs.org/E/2019/68 (accessed May 8, 2019).

[B61] Valero D. Redondo-Sama G. Elboj C. (2017). Interactive groups for immigrant students: a factor for success in the path of immigrant students. *Int. J. Incl. Educ.* 22 787–802. 10.1080/13603116.2017.1408712

[B62] van den Besselaar P. A. A. Flecha R. Radauer A. (2018). *Monitoring the Impact of EU Framework Programmes.* Brussels: European Commission 10.2777/518781

[B63] van der Veen C. de Mey L. van Kruistum C. van Oers B. (2017). The effect of productive classroom talk and metacommunication on young children’s oral communicative competence and subject matter knowledge: an intervention study in early childhood education. *Learn. Instruct.* 48 14–22. 10.1016/j.learninstruc.2016.06.001

[B64] Villardón-Gallego L. García-Carrión R. Yáñez-Marquina L. Estévez A. (2018). Impact of the interactive learning environments in children’s prosocial behavior. *Sustainability* 10:2138 10.3390/su10072138

[B65] Villarejo-Carballido B. Pulido C. M. de Botton L. Serradell O. (2019). Dialogic model of prevention and resolution of conflicts: evidence of the success of cyberbullying prevention in a primary school in catalonia. *Int. J. Environ. Res. Public Health* 16:918. 10.3390/ijerph16060918 30875775 PMC6466202

[B66] Vrikki M. Kershner R. Calcagni E. Hennessy S. Lee L. Hernández F. (2019a). The teacher scheme for educational dialogue analysis (T-SEDA): developing a research-based observation tool for supporting teacher inquiry into pupils’ participation in classroom dialogue. *Int. J. Res. Method Educ.* 42 185–203. 10.1080/1743727X.2018.1467890

[B67] Vrikki M. Wheatley L. Howe C. Hennessy S. Mercer N. (2019b). Dialogic practices in primary school classrooms. *Lang. Educ.* 33 85–100. 10.1080/09500782.2018.1509988

[B68] Vygotsky L. (1978). *Mind in Society: The Development of Higher Psychological Processes*. Cambridge, MA: Harvard University Press.

[B69] Wegerif R. (2011). Towards a dialogic theory of how children learn to think. *Think. Skills Creat.* 6 179–190. 10.1016/j.tsc.2011.08.002

[B70] Wegerif R. (2019). “Dialogic education,” in *Oxford Research Encyclopedia of Education*, ed. Noblit G. W. (Oxford: Oxford University Press). 10.1093/acrefore/9780190264093.013.396

[B71] Wegerif R. Doney J. Jamison I. (2017). Designing education to promote global dialogue: lessons from generation global—a project of the tony blair institute for global change. *Civitas Educ. Educ. Polit. Cult.* 6 113–129.

[B72] Wells C. G. (1999). *Dialogic Inquiry: Towards a Sociocultural Practice and Theory of Education.* Cambridge, MA: Cambridge University Press.

[B73] Wells G. Arauz R. M. (2006). Dialogue in the classroom. *J. Learn. Sci.* 15 379–428. 10.1207/s15327809jls1503_3

