# Supplementary material for: Implications for Social Impact of Dialogic Teaching and Learning
Source: Front Psychol. 2020 Feb 5;11:140. doi: 10.3389/fpsyg.2020.00140 (PMC7012899; doi:10.3389/fpsyg.2020.00140)
Supplement: Supplementary file 1 [file Table_1.DOCX]

Table 1. Principles of dialogic teaching (Alexander, 2018)

| **Principle** | **Definition** |
| --- | --- |
| Collective | The classroom is understood as a space of joint learning and enquiry |
| Reciprocal | Participants listen to each other, share ideas and consider alternative  viewpoints |
| Supportive | Participants feel able to express ideas freely, without risk of embarrassment over ‘wrong’ answers, and they help each other to reach common understandings |
| Cumulative | Participants build on their own and each other’s contributions and chain  them into coherent lines of thinking and understanding |
| Purposeful | Classroom talk, though open and dialogic, is structured with specific  learning goals in view |
