# Supplementary material for: Implications for Social Impact of Dialogic Teaching and Learning
Source: Front Psychol. 2020 Feb 5;11:140. doi: 10.3389/fpsyg.2020.00140 (PMC7012899; doi:10.3389/fpsyg.2020.00140)
Supplement: Supplementary file 2 [file Table_2.DOCX]

Table 2. Principles of dialogic learning (Flecha, 2000)

| **Principle** | **Definition** |
| --- | --- |
| egalitarian dialogue | Participants engage in dialogues with no coercion, manipulation or power interactions, drawing on validity claims |
| cultural intelligence | Every people knowledge is valued and included in schools and classrooms |
| transformations | Teaching and learning is oriented to overcome difficulties within the school community and beyond |
| instrumental learning | Learning is oriented towards achieving the maximum level of knowledge |
| creation of meaning | Dialogues are source of creation of meaning |
| solidarity | Participants engage in supportive and solidarity-based interactions where students feel share their viewpoints and thoughts |
| equality of differences | Diversity is an asset in the classroom and teaching and learning aim for equality of results valuing, respecting and building on the diversity in the class |
